# Age and sex modify the association between plasma Sestrin2 and bone mineral density: a cross-sectional study

**DOI:** 10.1093/jbmrpl/ziag127

**Published:** 2026-08-08

**Authors:** Muhammad Ammar Zahid, Hanan H Abunada, Ruaa Ahmed, Eiman Elamin, Hicham Raïq, Atiyeh M Abdallah, Abdelali Agouni

**Affiliations:** Department of Pharmaceutical Sciences, College of Pharmacy, QU Health, Qatar University, Doha, Qatar; Biomedical Research Center, QU Health, Qatar University, Doha, Qatar; Department of Pharmaceutical Sciences, College of Pharmacy, QU Health, Qatar University, Doha, Qatar; Department of Pharmaceutical Sciences, College of Pharmacy, QU Health, Qatar University, Doha, Qatar; Department of Social Sciences, College of Arts and Sciences, Qatar University, Doha, Qatar; Department of Biomedical Sciences, College of Health Sciences, QU Health, Qatar University, Doha, Qatar; Department of Pharmaceutical Sciences, College of Pharmacy, QU Health, Qatar University, Doha, Qatar

**Keywords:** Sestrin2, BMD, aging, osteoporosis, oxidative stress, biobank

## Abstract

Sestrin2 is a stress-inducible protein that plays a significant role in maintaining antioxidant balance and metabolic homeostasis via the AMPK/mTOR pathways. Animal models suggested that Sestrin2 deficiency may be associated with high bone mass; however, how this may translate into humans, especially in aging and stress conditions, is unknown. We performed a cross-sectional analysis of 910 adults recruited from the Qatar Biobank cohort. Participants were stratified into 3 groups: younger than 40 yr, 40-55 yr, and older than 55 yr. We investigated the relationship between plasma Sestrin2 and FN BMD T-score in adults using multiple linear regression, controlling for age, sex, BMI, diabetes, and estimated glomerular filtration rate. We observed a significant interaction between Sestrin2 and age (*p* < .001). In younger and middle-aged adults, Sestrin2 was not correlated with BMD. However, in adults aged >55 yr, Sestrin2 was strongly and positively correlated with FN T-scores (β = .131, 95% CI: 0.062-0.201, *p* < .001). This association remained significant after adjustment for all possible confounders, with an effect size reduction of only 7.7%. Further analysis showed that this association is more pronounced in males (β = .155, *p* < .001) but is not significant in females (β = −.008, *p* = .935). Our study indicates that Sestrin2 and BMD are significantly correlated but only in aged adults. In contrast to animal models, we showed that Sestrin2 in aged adults is positively correlated with bone density. This suggests that Sestrin2 may serve as a biomarker of resilience against aging-induced stress and osteosenescence. Furthermore, this resilience mechanism appears to be sex-specific, offering protective effects primarily in aging males, likely due to differences in hormonal preservation.

## Graphical Abstract

**Graphical Abstract:**
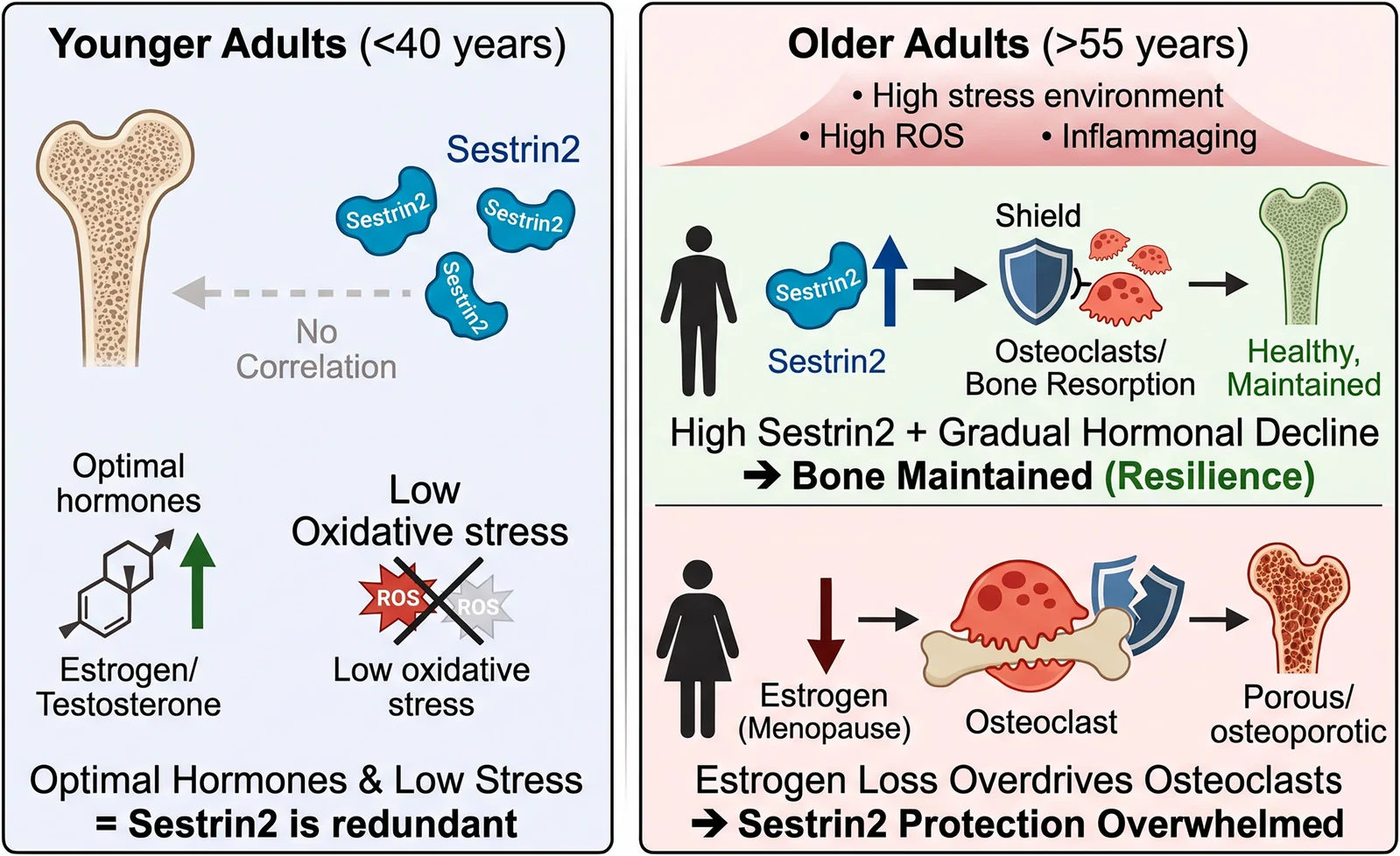

## Introduction

Osteoporosis is no longer considered solely an endocrine disorder of aging but also a systemic disorder of bone remodeling. Chronic, low-grade inflammatory processes, beginning with advanced age and oxidative stress, are now considered to play a pivotal role in the development of the disease, along with hormonal, genetic, and mechanical factors.^1–4^ In light of the projected multi-fold increase in the number of osteoporotic fractures worldwide by 2050, the focus is now on developing new approaches to the early, proactive identification of molecular and biochemical markers of the disease, in addition to the traditional DXA scan.^5^ This is of particular interest in the context of the high metabolic comorbidity observed in Qatar, where high BMI is associated with widespread vitamin D deficiency. In this demographic, traditional risk assessment tools are often confounded by the assumption that obesity protects against bone loss.^6–8^

The integrity of the skeleton is maintained by the balanced and coordinated processes of bone resorption and formation. Aging affects this process. Moreover, oxidative stress triggers an adipogenic switch in mesenchymal stem cells, leading to increased adiposity in the bone marrow and reduced osteogenesis, thus creating a pro-inflammatory feedback loop.^9^ To combat age-related injuries, cells use stress-inducible proteins, including Sestrin2. Sestrin2 is a highly evolutionarily conserved cysteine sulfinyl reductase and plays the central role in the interplay between metabolic and antioxidant signaling pathways.^10^^,^^11^ By controlling the overactive mTORC1 signaling pathway, which in turn accelerates senescence and suppresses autophagy in cells, Sestrin2 is thought to maintain the viability of long-lived osteocytes under metabolic stress.^12^

However, Sestrin2’s involvement in skeletal biology is also characterized by an interesting paradox. In Sestrin2 KO mice, high bone mass is consistently observed, due to defective osteoclast fusion and turnover, not increased bone formation,^13^ whereas Sestrin2 overexpression in cell models potently inhibits RANKL-induced osteoclastogenesis via AMPK activation and ROS suppression.^14^ Yet, as a stress-inducible protein, Sestrin2 levels in humans typically rise with age as a compensatory mechanism.^10^^,^^15^ This creates a critical knowledge gap: does elevated Sestrin2 in aging humans reflect pathological stress accumulation associated with bone loss, or a protective resilience response associated with bone preservation? Leveraging the extensive phenotyping of the Qatar Biobank (QBB), this study aims to resolve this paradox by establishing the association between plasma Sestrin2 and FN BMD in a diverse adult cohort. We specifically test the hypothesis that Sestrin2 acts as a conditional biomarker of resilience, becoming relevant to bone health only under the oxidative burden of biological aging.

## Materials and methods

### Study design and participants

This cross-sectional observational study used biospecimens and data obtained from the QBB, a large-scale, prospective population-based cohort initiated in 2012 for research purposes. The QBB recruited 60 000 adult Qatari nationals and long-term residents in Qatar (residing for at least 15 yr) and followed them for up to 5 yr.^16^ For the present cross-sectional analysis, data and stored plasma samples for 1000 participants were requested. These specific baseline clinical measurements and biological samples were collected by the QBB between 2016 and 2022. Participants underwent a comprehensive clinical assessment, including measurement of serum biomarkers and DXA scanning. The Institutional Review Boards (IRB) of QBB (E-2023-QF-QBB-RES-ACC-00119-0236) and Qatar University (QU-IRB 1993-EA/23) approved the study protocol. The study was also approved by the Qatar University Institutional Biohazard Committee (IBC) (QU-IBC-2022/032). Informed consent was obtained from all participants prior to data collection. The data provided by QBB was anonymized to protect participant confidentiality. Inclusion criteria were: (1) age ≥18 yr, (2) available plasma SESTRIN2 protein levels, and (3) DXA scan with FN T-score assessment. Exclusion criteria were: (1) current use of medications affecting bone metabolism (bisphosphonates, denosumab, teriparatide, and glucocorticoids), (2) known metabolic bone disease other than osteoporosis, (3) malignancy within the past 5 yr, and (4) incomplete key covariate data. The application of these criteria to the dataset requested from QBB resulted in a final sample of 910 participants.

### Laboratory measurements

Blood samples were collected after an overnight fast (≥8 h). Plasma was separated by centrifugation at 3000 × *g* for 10 min and stored at −80 °C until analysis. Plasma Sestrin2 concentrations were measured using an ELISA kit (SESTRIN2 ELISA Kit, BT LAB, Cat. No. E3437Hu; Bioassay Technology Laboratory), following the manufacturer’s protocol with slight modifications.^17^ The assay had a sensitivity of 0.01 ng/mL and a dynamic detection range of 0.05-15 ng/mL. Intra- and inter-assay coefficients of variation were kept below 8% and 10%, respectively. Standard biochemical parameters, including serum creatinine, glucose, and lipid profiles, were measured using automated analyzers. Estimated glomerular filtration rate (eGFR) was calculated using the CKD-EPI equation.^18^

### BMD assessment

Femoral neck BMD was assessed using DXA (GE Lunar iDXA), the standard technique for evaluating bone health. All scans were performed by certified technicians in accordance with standardized protocols. T-scores were calculated using manufacturer-provided reference databases comparing participant BMD to young adult mean values.

### Covariate assessment

Demographic information, medical history, and lifestyle factors were collected through standardized questionnaires. BMI was calculated as weight (kg) divided by height squared (m^2^). Diabetes status was defined as a self-reported physician diagnosis, use of antidiabetic medications, fasting glucose ≥126 mg/dL, or HbA1c ≥6.5%. Smoking status was categorized as current, former, or never smoker. Physical activity was assessed using the International Physical Activity Questionnaire (IPAQ). Menopausal status in women was determined by self-report and confirmed by clinical criteria (absence of menstruation for ≥12 mo and age >45 yr).

### Statistical analysis

#### Sample size and power

Based on preliminary data suggesting a moderate effect size (*f*^2^ = 0.10) for the Sestrin2 predictor in a multiple regression adjusted for 5 covariates (6 predictors total), 81 participants provide 80% power at α = .05 (2-sided); with *N* = 189, achieved power exceeded 99%.

#### Descriptive statistics

Continuous variables were tested for normality using the Shapiro–Wilk test. Normally distributed variables were presented as mean ± SD, while non-normally distributed variables were presented as median [IQR]. Categorical variables were presented as frequencies and percentages. Baseline characteristics were compared across age groups using the Kruskal–Wallis test for continuous variables and the Chi-square test for categorical variables.

#### Primary analysis

Participants were stratified into three age groups based on biological relevance and sample distribution: <40 yr (pre-middle age), 40-55 yr (middle age), and >55 yr (older adults). The primary outcome was FN T-score, and the primary predictor was plasma SESTRIN2 concentration. We first tested for effect modification by age by fitting a multiple linear regression model in the overall sample, including a Sestrin2 × age interaction term, adjusted for gender, BMI, diabetes status, and eGFR. Given the significant interaction (*p* < .001), we proceeded with age-stratified analyses. For each age stratum, we used multiple linear regression to assess the association between Sestrin2 (continuous variable, per 1 ng/mL increase) and FN T-score, adjusting for age (continuous, yr), gender (categorical: male, female), BMI (continuous, kg/m^2^), diabetes status (categorical: diabetic and non-diabetic), and eGFR (continuous, mL/min/1.73 m^2^).

Covariates were selected a priori based on established associations with bone health and were retained in models regardless of statistical significance to maintain consistent adjustment across analyses.

#### Secondary analyses

In the older adult subgroup (>55 yr), sensitivity analyses utilizing sequential adjustment analyses were performed to assess whether the SESTRIN2-BMD association was independent of potential confounders. We fitted 6 progressively adjusted models: (1) unadjusted, (2) age-adjusted, (3) additionally adjusted for gender, (4) additionally adjusted for BMI, (5) additionally adjusted for diabetes, and (6) fully adjusted (including eGFR). Effect attenuation was calculated as the percentage change in the SESTRIN2 coefficient from the unadjusted to the fully adjusted model. We tested for gender-specific effects in older adults by including a SESTRIN2 × gender interaction term in the fully adjusted model. Given a non-significant interaction (*p* > .05), we conducted exploratory gender-stratified analyses to generate hypotheses.

Further sensitivity analyses evaluated smoking status, physical activity level, serum calcium, corrected calcium, 25OHD, calcium supplement use, and vitamin D supplement use as potential additional covariates. Variables were considered confounders, if they were associated with both the outcome and the predictor in the older adult subgroup (*p* < .10). While 25OHD was statistically associated with both Sestrin2 (*p* < .001) and FN T-score (*p* = .051), including it as a covariate changed the Sestrin2 effect estimate by less than 1%, indicating negligible confounding. Neither calcium supplement use nor vitamin D supplement use was associated with Sestrin2 levels (*p* = .330 and *p* = .858, respectively) and including them changed the Sestrin2 coefficient by less than 5% and did not improve the model fit. Additionally, supplement data were available for only 49% and 67% of older adults, respectively. Smoking status, physical activity, calcium-related serum variables, and supplement use did not meet the criteria for confounding and were excluded from the final models to preserve statistical power and avoid overadjustment.

In older adult females (>55 yr), menopausal status was available for 100% of participants (*N* = 81). We conducted exploratory analyses to assess whether menopausal status modified the Sestrin2-BMD relationship. We tested for interaction using likelihood ratio tests comparing models with and without the Sestrin2 × menopause interaction term and performed stratified analyses by menopausal status when sample sizes permitted (*N* ≥ 10 for multivariable analysis). These exploratory analyses are presented as Table S2.

#### Model diagnostics

Model assumptions were assessed through examination of: (1) residual plots for homoscedasticity, (2) quantile–quantile plots for normality of residuals, (3) variance inflation factors (VIF) for multicollinearity (acceptable if VIF <5), and (4) Cook’s distance and leverage values for influential observations (flagged if Cook’s *D* > 0.5). Figure S1 presents diagnostic plots for the primary model.

#### Statistical software

All analyses were performed using Python 3.9 with pandas (version 1.3.5), scipy (version 1.7.3), statsmodels (version 0.13.2), matplotlib (version 3.5.1), and seaborn (version 0.11.2) libraries. A 2-sided *p*-value <.05 was considered statistically significant. No adjustments for multiple comparisons were made in the primary analyses, as hypotheses were specified a priori and analyses were hierarchically structured.

## Results

### Participant characteristics

Data and corresponding plasma samples for 1000 adult participants were initially requested and received from the QBB for this cross-sectional study. Upon reviewing, 90 participants were excluded based on our predefined criteria (eg, missing data on FN T-score or Sestrin2, history of malignancy, or use of bone-altering medications). After applying these exclusions, a final cohort of 910 participants was included in the analysis. Baseline characteristics for these 910 participants are presented in Table 1. The median age was 41 yr (IQR 31-52), with 446 participants <40 yr (49%), 275 between 40 and 55 yr (30%), and 189 >55 yr (21%). The cohort included 411 females (45%) and 499 males (55%). Median plasma Sestrin2 was 1.8 ng/mL (IQR 1.3-2.6), and median FN T-score was −0.5 (IQR −1.2 to 0.2). Median serum corrected calcium was 2.33 mmol/L (IQR 2.27-2.39), and median serum 25OHD was 16.0 ng/mL (IQR 11.0-23.0), with complete data available for 910 (100%) and 909 (99.9%) participants, respectively. Among participants with available supplement data, 11.2% (58/518) reported calcium supplement use, and 24.1% (143/594) reported vitamin D supplement use.

**Table 1:** Baseline characteristics stratified by age group.

Characteristic	Overall (*N* = 910)	Younger (*N* = 446)	Middle-aged (*N* = 275)	Older (*N* = 189)	*p*-value
**Age, yr**	41.0 [31.0-52.0]	31.0 [26.0-35.0]	48.0 [43.0-51.0]	62.0 [58.0-68.0]	<.001
**Sestrin2, ng/mL**	1.8 [1.3-2.6]	1.8 [1.3-2.8]	1.6 [1.3-2.3]	1.7 [1.3-2.3]	.011
**Femoral neck T-score**	−0.5 [−1.2 to 0.2]	−0.3 [−1.1 to 0.5]	−0.5 [−1.0 to 0.2]	−1.0 [−1.7 to 0.3]	<.001
**BMI, kg/m** ^**2**^	29.4 [26.1-33.4]	28.0 [24.3-32.2]	30.1 [27.4-34.1]	30.7 [27.7-35.4]	<.001
**eGFR, mL/min/1.73 m** ^**2**^	110.5 [98.4-119.6]	119.6 [113.1-125.3]	107.7 [100.2-112.2]	92.2 [65.7-100.1]	<.001
**25OHD, ng/mL**	16.0 [11.0-23.0]	13.0 [9.0-19.0]	16.0 [12.0-23.0]	23.0 [16.0–29.0]	<.001
**Serum calcium (corrected), mmol/L**	2.33 [2.27-2.39]	2.32 [2.25-2.37]	2.33 [2.27-2.38]	2.37 [2.31-2.43]	<.001
**Gender**					.108
**Male**	499 (54.8%)	229 (51.3%)	162 (58.9%)	108 (57.1%)	
**Female**	411 (45.2%)	217 (48.7%)	113 (41.1%)	81 (42.9%)	
**Diabetes status**					<.001
**Normal**	612 (67.3%)	387 (86.8%)	154 (56.0%)	71 (37.6%)	
**Diabetes**	298 (32.7%)	59 (13.2%)	121 (44.0%)	118 (62.4%)	
**Calcium supplement use**	58/518 (11.2%)	28/271 (10.3%)	15/154 (9.7%)	15/93 (16.1%)	<.001
**Vitamin D supplement use**	143/594 (24.1%)	47/288 (16.3%)	44/180 (24.4%)	52/126 (41.3%)	<.001

Data are presented as median [IQR] for non-normally distributed continuous variables and as *n* (%) for categorical variables. *p*-values were calculated using the Kruskal–Wallis test (continuous variables) and χ^2^ test (categorical variables). The *p*-values represent the overall differences across all 3 age groups rather than a comparison between any 2 specific groups. 25OHD was evaluated as a potential confounder but excluded from final multivariable models as it did not materially alter the Sestrin2–BMD association (effect change <1%). Abbreviation: eGFR, estimated glomerular filtration rate.

Significant differences were observed across age groups for all measured parameters except gender. Compared to the overall cohort, older adults (>55 yr) had lower Sestrin2 levels (median 1.7 vs 1.8 ng/mL, *p* = .011), lower FN T-scores (median −1.0 vs −0.5, *p* < .001), higher BMI (median 30.7 vs 29.4 kg/m^2^, *p* < .001), lower eGFR (median 92.2 vs 110.5 mL/min/1.73 m^2^, *p* < .001), higher serum corrected calcium (median 2.37 vs 2.33 mmol/L, *p* < .001), and higher serum 25OHD (median 23.0 vs 16.0 ng/mL, *p* < .001). Supplement use also varied by age, with older adults more likely to use both calcium (16.1% vs 11.2% overall) and vitamin D supplements (41.3% vs 24.1% overall).

### Age modifies the Sestrin2-BMD association

**Figure 1:**
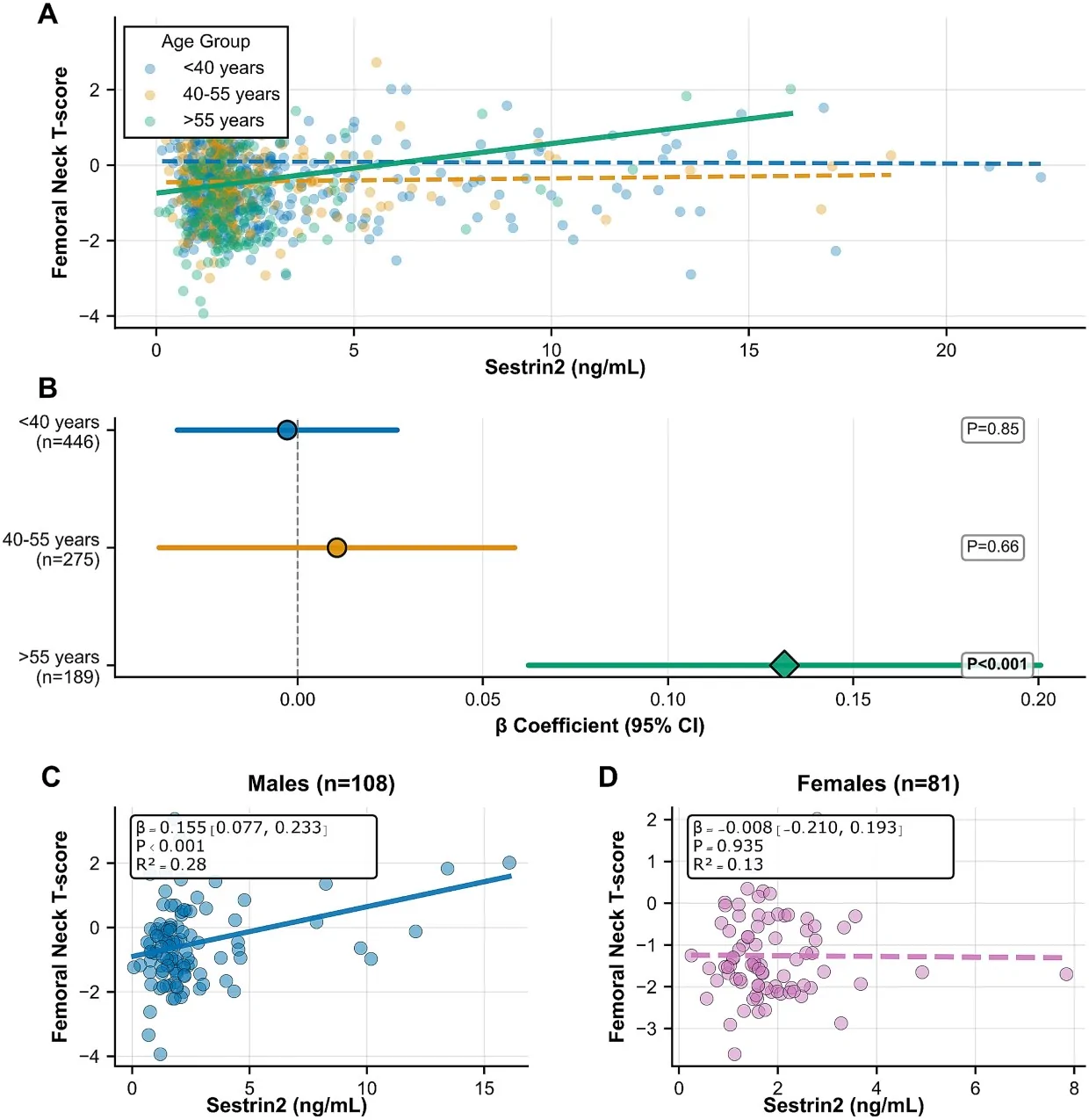
Association between plasma Sestrin2 levels and BMD by age and sex. (A) Scatter plot illustrating the correlation between plasma Sestrin2 (ng/mL) and FN T-score across three age cohorts (<40 yr, 40-55 yr, and >55 yr). The solid regression line for the oldest cohort indicates a distinct positive trend, whereas the dashed lines for the 2 younger cohorts are essentially flat. (B) Forest plot of β coefficients (95% CI) representing the association between Sestrin2 and FN T-score stratified by age. Models were adjusted for age, sex, BMI, diabetes status, and estimated glomerular filtration rate (eGFR). (C and D) Sex-stratified analysis within the >55 yr age group.

Effect modification by age was tested using an interaction analysis in the overall cohort. A multiple linear regression model adjusted for gender, BMI, diabetes status, and eGFR revealed a highly significant Sestrin2 × age interaction term (β = .0030, 95% CI: 0.0013-0.0048, *p* < .001), indicating that the relationship between Sestrin2 and FN T-score varies significantly with age (Table 2, Figure 1A).

**Table 2:** Association between Sestrin2 and FN T-score by age group.

Model	*N*	β Coefficient	SE	95% CI	*p*-value
**Interaction term (Sestrin2 × age)**	910	0.0030	0.0009	0.0013 to 0.0048	<.001
**<40 yr**	446	−0.003	0.015	−0.033 to 0.027	.851
**40-55 yr**	275	0.011	0.024	−0.037 to 0.059	.664
**>55 yr**	189	0.131	0.035	0.062 to 0.201	<.001

All models were adjusted for age, gender, BMI, diabetes status, and eGFR. The β coefficient represents the change in FN T-score per 1 ng/mL increase in Sestrin2.

Given the significant interaction, age-stratified analyses were performed (Table 2, Figure 1B). After adjustment for age, gender, BMI, diabetes status, and eGFR, there was no association between Sestrin2 and FN T-score (β = −.003, 95% CI: −0.033 to 0.027, *p* = .851) in <40 age group. The model explained 11.5% of the T-score variance (*R*^2^ = 0.115). Similarly, no association was observed in the 40-55 age group (β = .011, 95% CI: −0.037 to 0.059, *p* = .664, *R*^2^ = 0.229). In contrast, a strong positive association was found between Sestrin2 and FN T-score in the >55 age group. Each 1 ng/mL increase in plasma Sestrin2 was associated with a 0.131-point increase in T-score (95% CI: 0.062-0.201, *p* < .001). The fully adjusted model explained 29.2% of the T-score variance (*R*^2^ = 0.292).

### Independence from confounders

**Figure 2:**
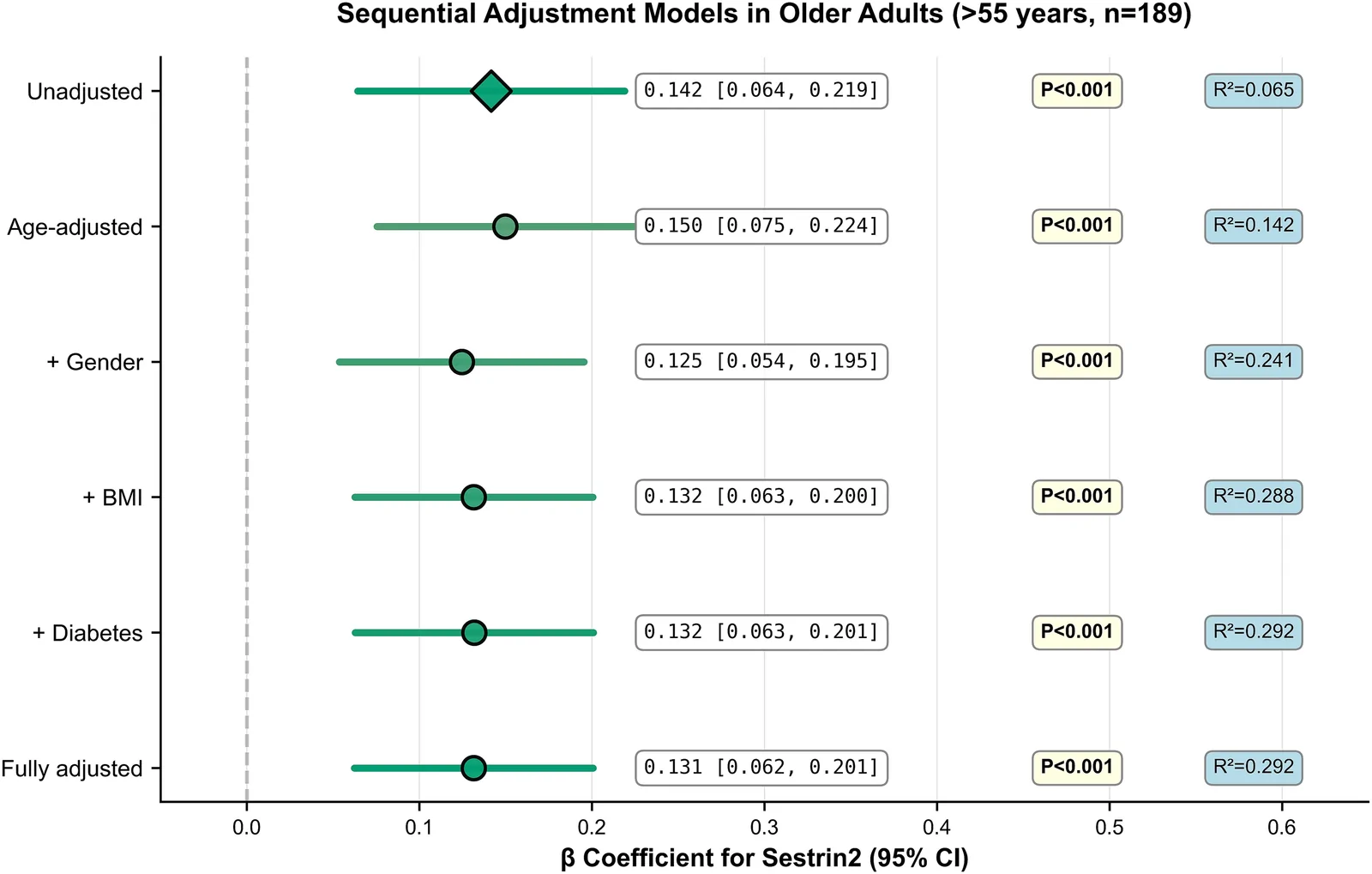
Sequential adjustment models for the association between Sestrin2 and BMD in older adults (>55 yr). Forest plot illustrating the robustness of the association between plasma Sestrin2 and FN T-score (*n* = 189) across various levels of adjustment. The fully adjusted model includes age, sex, BMI, diabetes status, and estimated glomerular filtration rate (eGFR).

To establish whether the Sestrin2-BMD association in older adults was independent of key confounders, sequential adjustment analyses were performed (Table 3, Figure 2). In unadjusted analysis, Sestrin2 showed a significant positive association with T-score (β = .142, 95% CI: 0.064-0.219, *p* < .001, *R*^2^ = 0.065). The association strengthened slightly after age adjustment, suggesting that age may have slightly suppressed the effect (β = .150, 95% CI: 0.075-0.224, *p* < .001, *R*^2^ = 0.142). Substantial increase in *R*^2^ was observed due to the strong independent effect of gender, but Sestrin2 effect remained stable, β = .125 (95% CI: 0.054-0.195, *p* < .001, *R*^2^ = 0.241). Adding BMI to the model had minimal change in the Sestrin2 coefficient (β = .132; 95% CI: 0.063-0.200, *p* < .001, *R*^2^ = 0.288), and adding diabetes status had no meaningful change (β = .132; 95% CI: 0.063-0.201, *p* < .001, *R*^2^ = 0.292). The fully adjusted model with eGFR had minimal attenuation of Sestrin2 effect, β = .131 (95% CI: 0.062-0.201, *p* < .001, *R*^2^ = 0.292). The Sestrin2 coefficient changed by only 7.7% from the unadjusted model (β = .142) to the fully adjusted model (β = .131), indicating robust independence from measured confounders. In the fully adjusted model, significant independent predictors of T-score were Sestrin2 (*p* < .001), age (*p* < .001), gender (*p* < .001), and BMI (*p* = .001), while diabetes status (*p* = .315) and eGFR (*p* = .768) were not significant.

**Table 3:** Sequential adjustment models for the association between Sestrin2 and FN T-score in older adults (>55 years, *N* = 189).

Model	β Coefficient	SE	95% CI	*p*-value	*R* ^2^
**Model 1: Unadjusted**	0.142	0.039	0.064-0.219	<.001	0.065
**Model 2: Age-adjusted**	0.150	0.038	0.075-0.224	<.001	0.142
**Model 3: + Gender**	0.125	0.036	0.054-0.195	<.001	0.241
**Model 4: + BMI**	0.132	0.035	0.063-0.200	<.001	0.288
**Model 5: + Diabetes**	0.132	0.035	0.063-0.201	<.001	0.292
Model 6: Fully adjusted*	0.131	0.035	0.062-0.201	<.001	0.292

Fully adjusted model includes age, gender, BMI, diabetes status, and eGFR. The β coefficient represents the change in FN T-score per 1 ng/mL increase in Sestrin2. Effect attenuation from Model 1 to Model 6: 7.7%, indicating minimal confounding. Abbreviation: eGFR, estimated glomerular filtration rate.

### Gender-stratified analysis

The association between Sestrin2 and BMD differed by gender in older adults. The Sestrin2 × gender interaction term in the fully adjusted model was not statistically significant (*p* = .147), suggesting no strong evidence for gender-specific effects at the conventional α = .05 threshold. However, exploratory gender-stratified analyses (Table S1, Figure 1C-D) revealed potentially different patterns. In males (*n* = 108), a strong positive association (β = .155, 95% CI: 0.077-0.233, *p* < .001, *R*^2^ = 0.28) was found, while in females (*n* = 81), no significant association was found (β = −.008, 95% CI: −0.210 to 0.193, *p* = .935, *R*^2^ = 0.13). Given the non-significant interaction test, we report the combined analysis as the primary result, with gender-stratified findings presented as hypothesis-generating observations requiring validation in larger cohorts.

### Menopausal status exploratory analysis

In older adult females (>55 yr), menopausal status data were complete for 100% of participants (*N* = 81), with 66 (81.5%) post-menopausal, 9 (11.1%) premenopausal, and 6 (7.4%) with hysterectomy (Table S2). We observed a borderline interaction between Sestrin2 and postmenopausal status (interaction *p* = .137, likelihood ratio test *p* = .116), suggesting potential effect modification. In stratified analysis, the postmenopausal group (*N* = 66) showed no significant association between Sestrin2 and T-score (β = −.055, 95% CI: −0.275 to 0.165, *p* = .620, *R*^2^ = 0.182), whereas the premenopausal group (*N* = 9) was too small for reliable multivariable analysis. Menopausal status was not independently associated with FN T-score after adjustment for age, BMI, eGFR, and diabetes.

### Covariate selection rationale and sensitivity analyses

We evaluated smoking status and physical activity level as potential additional covariates in the older adult subgroup. Neither variable was associated with FN T-score (smoking: *p* = .742; physical activity: *p* = .551) or with Sestrin2 levels (smoking: *p* = .765; physical activity: *p* = .304), indicating they did not meet criteria for confounding. Including both variables in the fully adjusted model resulted in no meaningful change in the Sestrin2 coefficient (β = .132 vs .131) but worsened model fit (AIC increased by 5.1 points). Therefore, these variables were not retained in the final models.

Similarly, we evaluated serum 25OHD as a potential confounder. While vitamin D was associated with both Sestrin2 (*p* < .001) and FN T-score (*p* = .051) in univariate analyses, including it as a covariate changed the Sestrin2 effect estimate by less than 1% (from β = .1314 to β = .1302), and did not improve model fit (LR test *p* = .212). This indicated a negligible confounding effect, and vitamin D was not included in the final models to preserve parsimony.

We also evaluated calcium and vitamin D supplement use as potential confounders. Supplement data were available for 49% of older adults (calcium) and 67% (vitamin D). Neither calcium supplement use nor vitamin D supplement use was associated with Sestrin2 levels (calcium: *p* = .330; vitamin D: *p* = .858) or FN T-score after adjustment (calcium: *p* = .096; vitamin D: *p* = .194). Including supplement use in models changed the Sestrin2 coefficient by less than 5% (calcium: 4.2%; vitamin D: 0.3%), with no improvement in model fit (likelihood ratio tests: calcium, *p* = .067; vitamin D, *p* = .165). Given the lack of confounding evidence and substantial missing data, supplement use was not retained in the final models.

### Model diagnostics

Diagnostic plots for the primary model (older adults, fully adjusted) are presented in Figure S1. Residuals showed no systematic patterns when plotted against fitted values, indicating homoscedasticity. The quantile–quantile plot demonstrated approximately normal distribution of residuals with no major deviations. All variance inflation factors were <3, indicating acceptable collinearity. Cook’s distance values were all <0.5, and no observations had excessive leverage, indicating results were not driven by influential outliers.

## Discussion

The main result of this study shows that Sestrin2 is positively associated with FN BMD in adults aged >55 years. In this respect, the study has redefined Sestrin2’s role from a stress-inducible regulator to a biomarker of bone health during aging. In younger adults (<40 yr old, median age 31), individuals are at or just completing the consolidation of peak bone mass. During this critical developmental window, bone density is heavily dictated by genetics and peak growth hormones, which naturally supersedes the influence of stress-inducible resilience pathways like Sestrin2. The skeletal system in this demographic is protected from severe oxidative stress and receives optimal hormonal support, making the compensatory upregulation of Sestrin2’s antioxidant functions unnecessary. However, with aging, the skeleton is exposed to a variety of increasingly challenging issues, including mitochondrial dysfunction, the formation of advanced glycation end-products, and low-grade chronic inflammatory processes (inflammaging). In this hostile environment, higher levels of Sestrin2 are critical for tissue survival, partly through Nrf2-mediated antioxidant mechanisms and the maintenance of constant AMPK levels.^14^

Our findings provide a much-needed human-centered perspective for the so-called “Sestrin2 Paradox.” In mouse models in which Sestrin2 is ablated, bone phenotype is characterized by increased bone mass due to reduced osteoclastogenesis and osteoclast fusion under standard developmental conditions.^13^ However, when we talk about aging in humans, we are no longer discussing developmental pathways; we are discussing pathways that involve oxidative stress. At a mechanistic level, Sestrin2 limits ROS-induced overactivation of osteoclasts via the NFATc1/TRAF6/p62 pathway and prevents adipocyte differentiation in bone marrow-derived mesenchymal stem cells. Therefore, in mice, the complete absence of Sestrin2 inhibits osteoclast fusion, thereby increasing bone mass. However, we argue that in aging humans, Sestrin2 is decreased to a degree that exacerbates oxidative stress and mitophagy-related problems. Therefore, in aging humans, an increase in Sestrin2 is an indicator of adaptive survival because it is an essential brake on ROS-mediated osteoclast formation, independent of its fundamental role in development.^19^^,^^20^

The loss of significant association in females might be due to sex differences in bone aging. In women, the onset of menopause causes a dramatic decline in estrogen levels, which triggers a hyperactive overdrive in osteoclast activity due to uncontrolled RANKL signaling and decreased cell death, beyond which Sestrin2 may be unable to protect against, regardless of how much is present.^21^^,^^22^ In contrast, in men, androgens decline more gradually, thereby safeguarding osteocytes and preserving the muscle–bone system. In men, Sestrin2 is abundant in skeletal muscle tissue and may be important in preventing sarcopenia. In addition, in older men, high systemic levels of Sestrin2 may help to preserve BMD not only by acting directly in bone but also by maintaining the mechanical load-bearing capacity of the muscle–bone system in sarcopenic osteopenia.^23^^,^^24^

These observations are important for people living in Qatar because many people in the country suffer from obesity and metabolic syndrome, making the usual risk assessment for fractures less effective. In such people, high BMI can actually mask underlying bone weakness on DXA scans because of the bone-loading effect of high body weight.^25^ The link between obesity and chronic low-grade inflammation is well established. Unlike creatinine or BMI, Sestrin2 is associated with cellular stress and is not dependent on kidney function or body size. Therefore, measuring Sestrin2 could potentially distinguish between “healthy” obese people and those with metabolic syndrome who are predisposed to fractures. It is crucial to acknowledge the high prevalence of diabetes (62.4%) in our older cohort. Patients with type 2 diabetes often exhibit a paradoxical bone phenotype characterized by normal or elevated BMD on DXA despite compromised bone microarchitecture and higher fracture risk. Therefore, Sestrin2 might interact with BMD differently in this highly diabetic population, highlighting the need for future studies evaluating bone quality alongside density. In theory, treatments that elevate or mimic Sestrin2 activity could potentially restore anti-osteoclast defense in these individuals by bolstering their resilience against metabolic and oxidative stress.^26^

It’s also important to recognize the limitations of the cross-sectional study design, as we cannot definitively demonstrate causality. Potential unmeasured confounding (such as dietary habits or genetic predispositions) and selection bias inherent to biobank volunteer cohorts may exist, which could potentially overestimate the observed associations. While we identify Sestrin2 as a candidate for a resilience pathway, longitudinal studies tracking incident fracture risk over time are required to determine whether elevated baseline levels of this protein truly predict the progression of bone loss. Our analysis also lacked data on hormone replacement therapy or testosterone replacement, which is a notable limitation given the sex-stratified findings. Additionally, while general self-reported physical activity (IPAQ) did not correlate with BMD in our modeling, this tool may not adequately capture specific osteogenic (bone-loading or resistance) activities. To tease apart differences in Sestrin2 signaling through the estrogen and androgen pathways, future studies will require detailed hormonal assessments by gender. While these findings are highly relevant to the Qatari demographic, their generalizability to other ethnicities or global populations requires further validation.

The present study provides a first hint that, in older individuals, Sestrin2 levels are positively correlated with FN bone density, whereas in younger individuals, no such correlation is observed. Exploratory analyses suggest this protective association is highly sex-specific, appearing predominantly in aging males rather than females. The age-dependent and risk-factor-independent nature of Sestrin2 suggests it as a new predictor of biological resilience. Older individuals—particularly men, who may benefit from more gradual hormonal decline—who are better at coping with the oxidative and metabolic challenges of their age by maintaining higher Sestrin2 levels also seem to be better at maintaining their bone density.

## Supplementary Material

Supplementary_ziag127

## Data Availability

Data are available from Qatar Biobank under a controlled-access process and cannot be publicly shared due to IRB and data-transfer restrictions (IRB: E-2023-QF-QBB-RES-ACC-00119-0236). Requests should be directed to QBB (qphi@qf.org.qa).
