# Supplementary material for: Age and sex modify the association between plasma Sestrin2 and bone mineral density: a cross-sectional study
Source: JBMR Plus. 2026 Aug 8;10(10):ziag127. doi: 10.1093/jbmrpl/ziag127 (PMC13537442; doi:10.1093/jbmrpl/ziag127)
Supplement: Supplementary file 1 [file supplementary_ziag127.docx]

## SUPPLEMENTARY DATA

**Supplementary Table S1.** Gender-Stratified Analysis of the Association Between Sestrin2 and Femoral Neck T-score in Older Adults (>55 Years)

| **Gender** | **N** | **β Coefficient (95% CI)** | **P value** |
| --- | --- | --- | --- |
| **Male** | 108 | 0.155 (0.077 to 0.233) | <0.001 |
| **Female** | 81 | −0.008 (−0.210 to 0.193) | 0.935 |

Models were adjusted for age, BMI, diabetes status, and eGFR. The β coefficient represents the change in femoral neck T-score per 1 ng/mL increase in Sestrin2. The interaction between Sestrin2 and gender was not statistically significant (P_interaction=0.147). CI, confidence interval; BMI, body mass index; eGFR, estimated glomerular filtration rate.

**Supplementary Table S2.** Exploratory Analysis of Menopausal Status Effect on the Association Between Sestrin2 and Femoral Neck T-score in Older Adult Females (N=81).

**A. Sample Characteristics by Menopausal Status**

| **Characteristic** | **Post-Menopausal (n=66)** | **Pre-Menopausal (n=9)** | **Hysterectomy (n=6)** | **Overall (n=81)** |
| --- | --- | --- | --- | --- |
| Age, years | 63.4 ± 6.2 | 59.1 ± 3.4 | 61.0 ± 4.1 | 62.8 ± 6.0 |
| BMI, kg/m² | 33.9 ± 7.2 | 32.4 ± 5.1 | 39.2 ± 10.8 | 34.1 ± 7.4 |
| Sestrin2, ng/mL | 1.89 ± 1.09 | 1.69 ± 0.90 | 1.75 ± 0.54 | 1.85 ± 1.03 |
| Femoral neck T-score | −1.31 ± 0.99 | −1.25 ± 0.93 | −1.36 ± 0.46 | −1.31 ± 0.95 |
| eGFR, mL/min/1.73m² | 77.3 ± 28.8 | 91.9 ± 33.0 | 93.4 ± 14.8 | 80.1 ± 28.8 |
| Diabetes, n (%) | 37 (56.1%) | 2 (22.2%) | 4 (66.7%) | 43 (53.1%) |

**B. Menopause × Sestrin2 Interaction Test**

| **Model** | **Sestrin2 β (P)** | **Post-menopause β (P)** | **Interaction β (P)** | **AIC** | **LR test P value** |
| --- | --- | --- | --- | --- | --- |
| Main effects | −0.0150 (0.884) | 0.1606 (0.563) | — | 223.14 | — |
| With interaction | 0.4504 (0.171) | 1.0640 (0.111) | −0.5164 (0.137) | 222.67 | 0.116 |

**C. Sestrin2–BMD Association Stratified by Menopausal Status**

| **Menopause Status** | **N** | **β (SE)** | **95% CI** | **P value** | **R²** |
| --- | --- | --- | --- | --- | --- |
| Post-Menopausal | 66 | −0.0548 (0.1099) | −0.275 to 0.165 | 0.620 | 0.182 |
| Pre-Menopausal | 9 | — | — | — | — |
| Hysterectomy | 6 | — | — | — | — |

All analyses were adjusted for age, BMI, eGFR, and diabetes status. Menopausal status data were available for 100% of older adult females (N=81). The interaction test compares models with and without the Sestrin2 × post-menopause interaction term using likelihood ratio testing. The pre-menopausal (N=9, 11%) and hysterectomy (N=6) groups were too small for reliable multivariable analysis. The borderline interaction (P=0.137) and opposite-direction effect in post-menopausal women warrant further investigation in larger studies incorporating direct hormone measurements.

**Supplementary Figure S1.** Regression Diagnostic Plots for the Association between Sestrin2 and Femoral Neck T-score in Older Adults.

**Alt text:** A two-by-two grid of four regression diagnostic scatter plots. Panel A, Residuals vs Fitted Values, shows data points randomly scattered around a horizontal dashed zero line with no clear pattern. Panel B, Normal Q-Q Plot, displays data points closely tracking a solid red diagonal line, with minor deviations at the extreme ends. Panel C, Scale-Location Plot, plots the square root of the absolute value of standardized residuals against Fitted Values, showing an even spread of points along a relatively flat red trend line. Panel D, Residuals vs Leverage, shows all data points clustered tightly along the horizontal zero line, well within the dashed red trumpet-shaped boundaries indicating Cook's distance.
